# GLIM-Defined Malnutrition in Patients with Head and Neck Cancer during the Qualification Visit for Home Enteral Nutrition

**DOI:** 10.3390/nu14030502

**Published:** 2022-01-24

**Authors:** Zuzanna Przekop, Magdalena Milewska, Dorota Szostak-Węgierek, Mariusz Panczyk, Jacek Sobocki

**Affiliations:** 1 Department of Clinical Dietetics, Faculty of Health Sciences, Medical University of Warsaw, 01-445 Warsaw, Poland; magdalena.milewska@wum.edu.pl (M.M.); dorota.szostak-wegierek@wum.edu.pl (D.S.-W.); 2 Department of Education and Research in Health Sciences, Faculty of Health Sciences, Medical University of Warsaw, 00-681 Warsaw, Poland; mariusz.panczyk@wum.edu.pl; 3 Department of General Surgery and Clinical Nutrition, Centre of Postgraduate Medical Education, 00-401 Warsaw, Poland; j.sobocki@mp.pl

**Keywords:** head and neck cancer, home enteral nutrition, nutritional status

## Abstract

Patients with head and neck cancer (HNC) present multiple symptoms that increase the risk of malnutrition. Nutritional care, including enteral nutrition (EN), plays a crucial role in the management of this group of patients. The aim of the study was to determine the Global Leadership Initiative on Malnutrition (GLIM)-based stages of malnutrition and the relationship with selected biochemical parameters during the home enteral nutrition (HEN) qualification visit of patients with HNC. The retrospective analysis involved 224 patients with HNC referred for HEN. The following parameters were evaluated: body mass index (BMI), percent weight loss, and laboratory tests (serum albumin, total serum protein, C-reactive protein (CRP), and total lymphocyte count (TLC)). Malnutrition was defined using GLIM-based criteria. The prevalence of malnutrition based on GLIM criteria was 93.75% (15.18% moderately malnourished, 78.57% severely malnourished). There was a positive correlation between malnutrition based on GLIM criteria, serum albumin, and CRP. In the model assessing the odds of severe malnutrition according to the criteria of GLIM, TLC and CRP had a statistically significant effect on the chance in the probability of qualifying a patient to the severe malnutrition group, but the strength of the results was weak. The prevalence of malnutrition in HNC patients enrolled to HEN is high and most of them are severely malnourished. This suggests that it is important to identify more efficiently patients with risk of malnutrition at an earlier stage. GLIM criteria for malnutrition can be easily applied in this group of patients, but the definition of inflammation criteria should be clarified.

## 1. Introduction

Malnutrition is one of the most common conditions in cancer diseases. A tumor in the advanced stage leads to increased catabolism and anorexia or even anorexia-cachexia syndrome composed of weight loss and negative shift in body composition [1,2,3]. Anorexia can be induced or aggravated by enhanced systemic inflammation that is related to cancer. Zeng et al. analyzed biochemistry-based indexes and their effect on toxicity. The authors reported that albumin and lymphocytes-monocytes ratio were independent prognostic factors for overall survival [4]. Thus, both inflammation and nutritional status have impact on the patient’s prognosis.

The prevalence of malnutrition varies depending on the tools used for its assessment [5,6]. In response to the global problem of malnutrition and the need for unification of its diagnosis, four major clinical nutrition societies joined together to create globally recognized criteria [7]. In 2018, after 3 years of work, the Global Leadership Initiative on Malnutrition (GLIM) published a recommendation for the diagnosis of malnutrition in adults [8]. As the criteria are relatively new, there is a lack of work based on them. Einarsson et al. suggested using GLIM criteria in nutrition research to provide multiple lines of evidence on the effects of malnutrition on treatment, prognosis, and survival [9]. The GLIM definition of malnutrition includes both phenotypic and etiologic groups of criteria, and it is necessary to meet at least one from each group to diagnose malnutrition [8].

In head and neck cancer (HNC), patients present multiple symptoms that increase the risk of malnutrition, such as dysphagia, xerostomia, poor appetite, or anorexia [10]. According to the European Society of Enteral Nutrition (ESPEN), regular assessment of food intake, weight, and body mass index (BMI) is recommended from the time of cancer diagnosis [2]. Due to the common nutrition-related problems and the occurrence of malnutrition, nutritional care plays a crucial role in the management of patients with head and neck cancer. Enteral nutrition is used in head and neck cancer patients due to tumor location, radiation therapy side effects, or inability to meet nutritional needs orally (energy intake, less than 60% of requirements for 1–2 weeks) [11,12,13].

The present study aimed to determine the relationship between the GLIM-defined malnutrition and the chosen biochemical parameters in patients with head and neck cancer referred to home enteral nutrition.

## 2. Materials and Methods

### 2.1. Study Design and Population

The study was designed as a retrospective analysis of medical records of all HNC patients referred to home enteral nutrition (HEN) in the reference national center in a scheduled period of time. The data were collected prospectively in the medical database in a systematic manner. All adult patients (≥18 years old) referred for home enteral nutrition between January 2018 and October 2021 who were diagnosed with cancer of the head or neck were included in the study. All patients got the referral to HEN from GP or another doctor and signed the consent of inclusion for the HEN procedure. Patients with incomplete medical records missing required for this study were excluded from the study. A group of 224 patients were included in the analysis. The study was approved by the Ethics Committee of the Medical University of Warsaw (KB/87/2018). 

### 2.2. Institutional Standard to Qualification for Home Enteral Nutrition

Routine institutional practice to qualify for home enteral nutrition includes blood tests, medical checkups, and nutrition evaluation. Blood tests include blood cell morphology and liver function tests include alanine aminotransferase (ALT), asparate aminotransferase (AST), alkaline phosphatase (ALP), lactate dehydrogenase (LD), bilirubin, gamma-glutamyltransferase (GGT), total serum protein, serum albumin, C-reactive protein, venous ionogram (potassium, sodium, calcium, magnesium, phosphorus, chlorides), urea and creatinine, glucose, total cholesterol, triglycerides, and coagulation factors.

#### Anthropometric Measurements

Nutritional assessment were run by trained dietitians or nurses and included anthropometric measurements (height, body weight). Body weight was measured according to the recommendations of Centers for Disease Control and Prevention CDC, using an electric scale (Fawag S.S. model ZOL-3.4. w. WTL, Lublin, Poland) with an accuracy of 0.1 kg [14]. Height was declared by patients.

### 2.3. Data Collection

All data were abstracted from the medical records. Collected data included demographic data (gender, age at HEN qualification, tumor localization), weight, height, unintentional weight loss in 6 months, and laboratory results. 

#### Nutritional Assessment

Body mass index (BMI) was calculated by dividing weight (kg) by height (m) squared. BMI categories were classified according to the criteria of CDC: underweight (BMI < 18.5 km/m^2^), normal weight (BMI ≥ 18.5 kg/m^2^–< 25.0 kg/m^2^), overweight (BMI ≥ 25.0 kg/m^2^) [14], and the criteria established by Lipschitz for the elderly population (≥65 years), with a range of normal values of 22–27 kg/m^2^ [15]. The percentage of weight loss in 6 months was calculated according to the following formula:

(1) $$\%\;\mathrm{of}\;\mathrm{weight}\;\mathrm{loss}=\frac{\mathrm{weight}\;\mathrm{loss}\;\left(\mathrm{kg}\right)}{\mathrm{current}\;\mathrm{weight}\;\left(\mathrm{kg}\right)+\;\mathrm{weight}\;\mathrm{loss}\;\left(\mathrm{kg}\right)}\times 100$$

Based on the collected data, the GLIM criteria were used to assess the nutritional status of patients [8]. Since the muscle mass reduction data were not available prior to qualification visit, in the case of phenotypic GLIM criteria, only weight loss and BMI were assessed. The dysphagia and the inflammation were defined as etiologic GLIM criteria, and together with the presence of metastatic disease or reduced food intake, they were defined as the referral to enteral nutrition. The overview of how the GLIM Criteria for the Diagnosis of Malnutrition were used in the present study are shown in Table 1.

The severity of malnutrition was then assessed as follows. Moderate malnutrition was diagnosed if any of the following criteria were met: weight loss < 5% within the last 6 months or BMI < 20 kg/m^2^ if aged < 70 years or <22 kg/m^2^ if aged ≥ 70 years. Severe malnutrition was diagnosed if weight loss > 5% within the last 6 months or BMI < 18.5 kg/m^2^ if <70 years of age or <20 kg/m^2^ if the patient was ≥70 years of age.

### 2.4. Laboratory Data

For the purpose of the present study, the following biochemical data were used: serum albumin, total serum protein, C-reactive protein, and total lymphocyte count (TLC). TLC was determined according to the following formula: (% lymphocytes × leukocytes)/100. The cut-off values for total lymphocyte count used for classification of immunological depletion were: <800 cells/mm^3^—severe depletion, 800–1999 cells/mm^3^—moderate depletion, and >2000 cells/mm^3^—no immunological depletion [16]. To evaluate malnutrition based on serum albumin, the following criteria were used: >3.5 g/dL—nourished, 3.0–3.5 g/dL—mild malnutrition, 2.4–2.9 g/dL—moderate malnutrition, and <2.4 g/dL—severe malnutrition. According to ESPEN guidelines, CRP levels < 10.0 mg/dL were considered normal [2]. However, to get a better overview, we also decided to use categorization according to Pourhassan et al.: <0.49 mg/dL no inflammation; 0.5–3.0 mg/dL mild inflammation; ≥3 mg/dL inflammation [17]. For the total serum protein, the laboratory reference values were used. The range 6.20–8.30 g/L was considered normal.

### 2.5. Statistical Analysis

Quantitative and categorical variables are described with descriptive statistical methods. For quantitative variables, the following measures were determined: central tendency (mean, M) and dispersion (standard deviation, SD). For the categorical variables, the following measures were determined: Number (*n*) and frequency (%).

Cross-tables and the Pearson’s chi-squared test were used to assess frequency difference for variants of categorical variables. To assess the impact of the sex and age group on the selected quantitative variables, Student’s *t*-test was used. The assessment of the relationship between the selected factors (tumor site, GLIM groups) and the value of quantitative variables was performed using one-way ANOVA with post hoc analysis by Fisher’s Least Significant Difference test. The discriminant ability of selected quantitative variables (albumin, TLC, CRP, and total protein) to differentiate between GLIM groups (moderate vs. severe status) was estimated using Receiver Operating Characteristic (ROC) curves.

A model of non-linear estimation for the logistic regression for assessing the odds of severe malnutrition with GLIM criteria was tested. Rosenbrock and a quasi-Newton method of estimation were applied, appointing asymptotic standard errors. For each predictor, the odds ratio (OR) was determined, together a with 95% confidence interval.

All calculations were performed with STATISTICA 13.3 software (TIBCO Software, Palo Alto, CA, USA). For all analyses, a *p*-level of <0.05 was considered statistically significant.

## 3. Results

### Group Characteristics

A total of 237 patients met the eligibility criteria of head and neck cancer diagnosis and enrolling to qualify a visit for HEN after referral to this procedure. Among them, 224 had complete data for the statistical analysis scheduled in the study. The mean age of patients was 62.69 ± 11.02 years, 24.5% were female, and 55.4% of them were below 65 years of age. The mean BMI was 20.77 ± 4.12 kg/m^2^ and the mean weight revealed 60.20 ± 13.39 kg. Mean BMI was significantly higher in the elderly (≥65 years) population (21.41 kg/m^2^ vs. 20.26 kg/m^2^, *p* < 0.05). The characteristics of the group, divided by sex and age, are shown in Table 2. The mean loss of body mass in the last 6 months was 17.03 ± 9.58%. The prevalence of BMI categories showed no significant difference between males and females, while there was a significant difference between the under 65 and elderly group (χ^2^df = 2 = 14.530, *p* = 0.001). Underweight was diagnosed in 34.68% of patients under 65 years of age and in 60% of elderly patients, while overweight was diagnosed in 12.10% and 9.0%, respectively.

The distribution of cancer types was as follows: 32.6% (*n* = 73) oral cavity, 30.8% (*n* = 69) oropharynx, 17.0% (*n* = 38) larynx, 12.1% (*n* = 27) hypopharynx, 3.6% (*n* = 8) nasopharynx, 1.8% (*n* = 4) nasal cavity and paranasal sinuses, 0.9% (*n* = 2) salivary glands, and 1.3% (*n* = 3) other types. In the group of patients with oropharynx cancer, 33 had a tonsil cancer (14.7% of total group). There were no statistically significant differences in tumor localization in age and sex groups.

**Figure 1:**
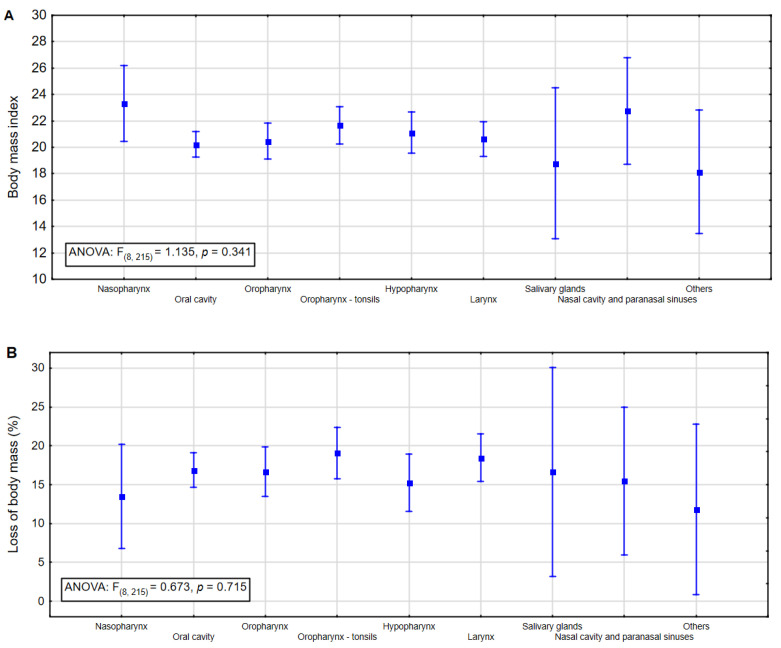
(**A**) Differences in mean of BMI divided into tumor site; (**B**) differences in mean of % of body weight loss in the last 6 months divided into tumor sites. The midpoint is the mean value, and the vertical line represents the 95% confidence interval.

The differences in BMI and percent body weight loss in the 6 months before qualification to home enteral nutrition (mean and confidential 95% interval), divided by tumor site, are shown in Figure 1. There was no statistical difference in these analyzes, so no post-hoc analysis was performed.

Table 3 shows the summary of body weight loss and biochemical data used in the further analysis.

Based on that used in the study GLIM criteria, 100% of the group met the etiologic criteria, 90.63% of patients demonstrated unintentional weight loss criteria, and 46.0% had low body mass index. There was no significant difference in the prevalence of those two phenotypic criteria between men and women or according to tumor location. Moderate malnutrition was diagnosed in 15.18% and severe malnutrition in 78.57% of whole group. The prevalence of malnutrition and its stages are presented in Table 4.

The summaries of biochemical data used for further analysis: serum albumin, total lymphocyte count, CRP, and total serum protein in patients with head and neck cancer receiving home enteral nutrition due to the cancer, divided into GLIM-nutritional stages, are presented in Table 5.

**Figure 2:**
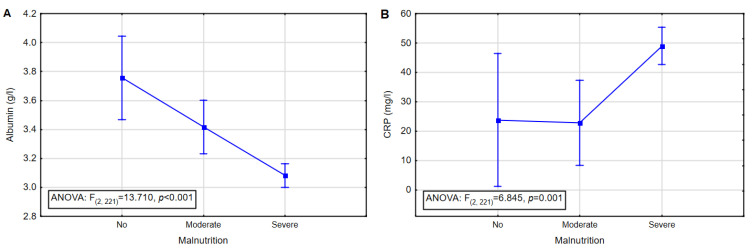
One-way ANOVA results of Albumin and CRP in HNC patients. (**A**) Albumin; (**B**) CRP. The midpoint is the mean value, and the vertical line represents the 95% confidence interval.

The one-way analysis of variance was used for laboratory data to determine whether there was a statistically significant difference between GLIM groups. A significant difference (using Fisher’s Least Significant Difference test) was found for serum albumin (F = 13.710, *p* = 0.000) and CRP (F = 6.845, *p* = 0.001), as shown in Figure 2. In the case of both parameters, there was a statically significant difference between no malnourished patients and those with severe malnutrition (serum albumin *p* < 0.001, CRP *p* < 0.05) and between those with moderate and severe malnutrition (serum albumin *p* = 0.0013, CRP *p* = 0.0013). 

The strength of association between defined by GLIM criteria of malnutrition and laboratory data was measured. In the case of albumin, there was a statistically significant positive correlation (z = 4.666, *p* < 0.001). The ROC analysis results of chosen laboratory data are presented in Table 6 and Table 7. The diagnostic parameters of severe malnutrition diagnosis were calculated for albumin (cutoff point—3.3 g/L) and CRP (cutoff point—32.62 mg/L). 

A logistic regression model for assessing the odds of severe malnutrition with GLIM criteria was constructed. The following variables were included in the model: age, percentage of body weight, WBC, total protein, albumin, CRP, and TLC.

Two predictors had a significant effect on the change in the probability of qualifying a patient being classified in the severe malnutrition group. In the case of TLC, the higher the value, the greater the chance of positive classification, and for each unit increase in TLC, the chance increased by 0.1% (*p* = 0.024, OR = 1.00, 95% CI 1.00 to 1.0001). In the case of CRP, the higher the value, the greater the chance of positive classification and for each unit increase in CRP, the chance increased by 3% (*p* = 0.001, OR = 1.03, 95% CI 1.012 to 1.055).

## 4. Discussion

One of the common consequences of head and neck cancer is malnutrition caused by the cancer and treatment complications, such as dysphagia, xerostomia, odynophagia, thick saliva, or mucositis [10,18]. The exact prevalence of malnutrition in head and neck cancer varies and depends on the stage of neoplastic disease, the treatment used, and criteria applied for malnutrition diagnosis. Citak et al. reported that the prevalence of malnutrition was 10% at the time of diagnosis of head and neck cancer and 74% at the end of radiotherapy (used tool: Scored Patients-Generated Subjective Global Assessment (PG-SGA)) [19]. In our study, the prevalence of malnutrition, during the qualification visit for home enteral nutrition, according to BMI value, was 46.0%, while the percentage of overweight patients was 10.7% and the prevalence of malnutrition, according to the GLIM criteria, in the whole group was 93.75%, which was higher than the results of other authors. Moreover, we observed a trend in the relationship between sex and malnutrition. The prevalence of malnutrition was more frequent in the male group, but there was no significant difference (*p* = 0.173), which could be due to the relatively small group of non-malnourished patients. The NutriCancer study, conducted in 2005 in 154 French hospitals, showed that only 50% of patients with head and neck cancer were malnourished. Malnutrition definition in this study was based on BMI [20]. Similar results based on body mass index were reported by Righini et al., according to whom 49% of patients with head and neck cancer were malnourished, and the prevalence of malnutrition was highest in patients with cancer of the oropharynx and oral cavity [21]. In our study, 75% of patients with oral cavity cancer and 80% with oropharynx cancer were severely malnourished, but there was no significant difference in the prevalence of stage of malnutrition, according to tumor location. According to our analysis, the mean percentage of unintentional body weight loss at the time of qualification for home enteral nutrition was 17.03% (SD = 9.53). The results reported in the systematic review by Bak et al. showed that the median weight loss was lower at 10.66% (3–25.5%), which corresponded to 7.5 kg (3–10.7 kg) vs. 12.62 kg (min 0.00 kg, max 40.00 kg, CV 63.2%) in our study [22]. Similar to our results, Einarsson et al. reported that weight loss was the most common phenotypic GLIM criterion [9]. Patients were involved in our study at the time of onset of the HEN procedure and, to our best knowledge, there was no similar research before, so results comparison is difficult. However, enteral nutrition is recommended supportive therapy for HNC, and we realize that our group might be more advanced in symptoms and not representative for a general group of HNC patients. Nonetheless, our results suggest that the referral to HEN should be made earlier. Unintentional weight loss in cancer patients is associated with poorer prognosis, independently of treatment, and may affect vitality and increase the prevalence of treatment side effects [23,24]. The ESPEN guidelines recommend that nutritional status be assessed regularly to detect nutritional disorders early. On the other hand, yearly published data show that only 20% of hospitals in Poland that participated in the nDay Survey recorded their patients’ weight on a weekly basis [25]. It is important that national healthcare regulations force the use of one malnutrition detection tool at every hospital admission. 

In our study, the analyzed laboratory data showed that the mean value of serum albumin was 3.18 g/dL (SD = 0.58), which was similar to the results obtained by Magnano et al., performed on patients with head and neck cancer (3.19 g/dL) [26]. Notably, 159 (76%) patients in our study had a low value of serum albumin (<3.5 g/dL). Similar to our results, Gascon-Ruiz et al. showed that there is a strong association between GLIM criteria and serum albumin in cancer patients, but in the study by Gascon-Ruiz et al., the proportion of patients with head and neck cancer was only 11.5% of the total study group [27]. Our results show that the cutoff for severe malnutrition was 3.3 g/dL, but the strength of the result was weak. Other studies suggest that a low serum albumin level may be a prognostic factor for 1-year mortality and postoperative complications in patients with head and neck cancer [28,29]. It should be pointed out that serum albumin has only the prognostic value of malnutrition prevalence. There is an association between serum albumin level and malnutrition, but it cannot be used as a nutritional marker. 

We also found an association between malnutrition and the inflammatory process, which is usually accompanied by negative nitrogen balance [30]. This seems to be confirmed in our analysis, where only TLC and CRP have a significant effect on changing the probability of a patient being classified in the severe malnutrition group. For TLC, the strength of correlation was weak, and the direction of correlation was the opposite to our expectation. The result may be caused by the criteria chosen, and the three cut-off values proposed by Calixto-Lima may influence poor data matching [16]. Our results may also be disturbed by possible coexisting infections, such as bacteriemia and viral or fungal infections [31,32]. 

As in our study, the inflammation was defined as the presence of metastatic disease and the correlation with serum CRP was performed. Surprisingly, the results of the diagnostic parameters for serum C-reactive protein did not meet the criteria for validation recommended by Schuener et al. and Keller at al. [7,33]. Some authors suggest that cancer should not be used as a positive criterion for inflammation and that serum CRP should be determined additionally [9]. According to the criteria of GLIM and current knowledge, malignancy is associated with chronic disease-related inflammation, and C-reactive protein is suggested as a supportive laboratory measure. In clinical practice, these criteria may be sufficient, but for research purposes and to build a consistent evidence base on nutritional status, the GLIM definition of inflammation should be clarified. In addition, all patients referred for HEN met the criteria for reduced food intake. The reason for qualification (i.e., inability to meet nutritional needs) may also have an impact on the CRP level. On the other hand, some authors reported that in patients with inflammation, the CRP level is not suitable for monitoring nutritional support [34]. ESPEN guidelines indicate that patients with low cancer activity and no inflammatory response, defined as serum CRP < 10 mg/dL, should receive nutritional support because their nutritional status can deteriorate rapidly [2]. The inflammatory criteria defined by serum CRP level can inadvertently delay the decision of nutritional intervention.

The study was conducted in a national reference center of HEN. A strength of our analysis is that it is one of the first cross-sectional studies accessing patients with head and neck cancer at the qualification to home enteral nutrition and first used GLIM criteria in this group of patients. However, this was a retrospective analysis: only 5% of patients dropped out of the study due to medical records that did not meet criteria. It shows that the GLIM criteria can be readily applied to patients on home enteral nutrition. 

## 5. Limitations

Our study had some limitations. Firstly, there was no information about the stage of cancer or the cancer treatment used in the available medical records. Secondly, due to the retrospective design of the study, it was impossible to assess the reduction in muscle mass and weight loss beyond 6 months. Thirdly, because of the lack of clearly defined and standardized information on cutoff values for biochemical data, we had to define our criteria based on other authors’ research, but there are more ways to define those criteria. 

## 6. Conclusions

This study highlights that the patients enrolled in the home enteral nutrition procedure are malnourished and most of them are severely malnourished. This suggests that it is important to more efficiently identify patients at risk of malnutrition so that appropriate and sufficient nutritional management can be implemented. Our findings suggest that GLIM criteria of malnutrition can be easily applied for groups of patients on home enteral nutrition, but the definition of inflammation criteria should be clarified for patients with head and neck cancer. Based on our results, a serum CRP of 32.62 mg/L seems to be an appropriate threshold of inflammation for severe malnutrition. 

## Figures and Tables

**Table 1:** Overview of how the GLIM Criteria for the Diagnosis of Malnutrition were used in the present study.

Group of GLIM Criteria	Description of GLIM Criteria	Criteria Used in the Study
Phenotypic	Weight loss	
	>5% within past 6 months	Present weight compared to self-estimated weight 6 months earlier
	>10% beyond 6 months	Not available
	BMI ^1^ (kg/m^2^)	
	<20 if <70 years	<20 if <70 years
	<22 if ≥70 years	<22 if ≥70 years
	Reduced Muscle Mass	
	FFMI ^2^ < 17 (males)	Not available
	FFMI < 15 (females)	
Etiologic	Reduced food intake	
	≤50% for >1 week, or any reduction for >2 weeks	Artificial (enteral) nutrition ^3^
	Inflammation	
	Acute disease/injury or chronic disease-related	Head and neck cancer ^2^

^1^ Body mass index. ^2^ Fat free mass index, FFMI (kg/m^2^). ^3^ Inclusion criteria to the study.

**Table 2:** Group characteristics of the study group divided by sex and age.

Characteristics	Female (*n* = 55)		Male (*n* = 169)		*p*-Value *	MD (95% CI)	<65 (*n* = 124)		≥65 (*n* = 100)		*p*-Value *	MD (95% CI)	Total	
	M	SD	M	SD			M	SD	M	SD			M	SD
Age	62.49	11.49	62.76	10.90	0.98	−0.27 (−3.65; 3.11)	NA	NA	NA	NA	NA	NA	62.69	11.02
Body mass (kg)	52.73	12.58	62.64	12.77	<0.001	−9.91 (−13.80; −6.01)	59.60	12.29	60.20	13.39	0.55	−1.36 (−4.91; 2.19)	60.20	13.39
Height (m)	1.59	0.06	1.73	0.07	<0.001	−0.134 (−0.16; −0.11)	1.71	0.09	1.68	0.09	0.01	0.03 (0.00; 0.06)	1.70	0.09
BMI kg/m^2^	20.58	4.62	20.83	3.96	0.77	−0.25 (−1.52; 1.01)	20.77	3.90	20.77	4.12	0.04	−1.15 (−2.23; −0.07)	20.77	4.12
Loss of body mass (%)	16.19	10.90	17.30	9.13	0.55	−1.11 (−4.05; 1.82)	17.03	9.22	17.03	9.58	0.88	0.36 (−2.19; 2.90)	17.03	9.58

M—mean, SD—standard deviation, MD—difference in means, CI—confidence interval, NA—not analyzed * Student’s *t*-test.

**Table 3:** Body weight loss and laboratory data of HNC patients (*n* = 224).

Characteristic	M	SD	Minimum	Maximum	CV (%)
Body weight loss (kg)	12.62	7.98	0.00	40.00	63.2
Leukocytes (thousand/mm^3^)	10.59	7.77	2.63	64.43	73.4
Lymphocytes (%)	13.64	8.67	0.30	48.00	63.6
Total protein (g/L)	7.43	6.06	2.70	97.00	81.6
Albumin (g/L)	3.18	0.58	1.40	4.50	18.2
CRP (mg/L)	43.45	43.99	1.34	256.98	101.2
Total lymphocyte count (cells/mm^3^)	1224.99	792.85	35.37	5202.01	64.7

**Table 4:** Prevalence of malnutrition based on GLIM criteria divided by sex, tumor site, and age.

	No Malnutrition		Moderate Malnutriton		Severe Malnutrition		*p*-Value *
	*n*	%	*n*	%	*n*	%	
Sex							0.173
Female	6	42.9	10	29.4	39	22.2	
Male	8	57.1	24	70.6	137	77.8	
Tumor Site							0.225
Nasopharynx	2	14.3	0	0.0	6	3.4	
Oral cavity	1	7.1	17	50.0	55	31.3	
Oropharynx	3	21.4	5	14.7	28	15.9	
Oropharynx-tonsils	3	21.4	3	8.8	27	15.3	
Hypopharynx	3	21.4	6	17.7	18	10.2	
Larynx	2	14.3	3	8.8	33	18.8	
Salivary glands	0	0.00	0	0.00	2	1.1	
Nasal cavity and paranasal sinuses	0	0.00	0	0.00	4	2.3	
Others	0	0.00	0	0.00	3	1.7	
Age							0.948
<65	8	57.1	18	52.9	98	55.7	
≥65	6	42.9	16	47.1	78	44.3	

* Pearson’s chi-squared test.

**Table 5:** Summary of biochemical data used in correlation with the stage of malnutrition of HNC patients.

GLIM Category		M	Mdn	Min	Max	SD
No malnutrition (*n* = 14)	Albumin (g/L)	3.8	3.9	3.1	4.5	0.43
	Total lymphocyte count (cells/mm^3^)	1472	1220	395	3760	1034
	CRP (mg/L)	23.8	9.9	1.85	94.72	31.03
	Total protein (g/L)	7.4	7.3	5.9	9.0	0.75
Moderate malnutrition (*n* = 34)	Albumin (g/L)	3.4	3.5	2.5	4.2	0.42
	Total lymphocyte count (cells/mm^3^)	1012	964	146	2042	547
	CRP (mg/L)	22.9	13.5	2.02	94.82	23.21
	Total protein (g/L)	7.20	7.2	5.6	8.4	0.67
Severe malnutrition (*n* = 176)	Albumin (g/L)	3.1	3.2	1.4	4.4	0.58
	Total lymphocyte count (cells/mm^3^)	1247	998	35	5202	807
	CRP (mg/L)	49.0	34.0	1.34	256.98	46.35
	Total protein (g/L)	7.5	7.0	2.7	97.0	6.83

Mdn—Median.

**Table 6:** ROC analysis results of the biochemical data investigated in the study as predictors of GLIM-based malnutrition.

ROC Results	Albumin	TLC	CRP	Total Protein
AUC (CI 95%)	0.672 (0.58–0.77)	0.431 (0.32–0.54)	0.701 (0.61–0.79)	0.582 (0.48–0.69)
SE	0.049	0.056	0.047	0.054
z	3.472	−1.233	4.292	1.507
*p*	0.0005	0.218	0.0000	0.132
Sensitivity (95% CI)	67% (59% to 73%)	NA	52% (45% to 60%)	NA
Specificity (95% CI)	62% (44% to 78%)	NA	82% (65% to 93%)	NA
Positive Predictive Value (95% CI)	90% (85% to 93%)	NA	94% (88% to 97%)	NA
Negative Predictive Value (95% CI)	26% (20% to 33%)	NA	25% (21% to 29%)	NA
Accuracy (95% CI)	66% (59% to 72%)	NA	57% (50% to 64%)	NA

NA—not analyzed, ROC—receiver operating characteristic, AUC—area under curve, CI—confidence interval, SE—standard error, TLC—total lymphocyte count, CRP—C-reactive protein.

**Table 7:** Result of Goodman Kruskal’s gamma correlation of biochemical data.

GLIM Category		GLIM Moderate Malnutrition		GLIM Severe Malnutrition		γ *	z	*p*-Value
		*n*	%	*n*	%			
Albumin	Nourished	15	44.1	36	20.5	0.51	4.666	<0.001
	Mild malnutrition	16	47.1	90	51.1			
	Moderate malnutrition	3	8.8	29	16.5			
	Severe malnutrition	0	0.0	21	11.9			
TLC	No depletion	1	2.9	23	13.1	−0.36	−3.072	0.002
	Moderate depletion	17	50.0	98	55.7			
	Mild depletion	16	47.1	55	31.3			
CRP ^1^ [17]	Mild inflammation	2	5.9	2	1.1	0.69	2.757	0.006
	Severe inflammation	32	94.1	174	98.9			
CRP ^2^ [2]	No inflammatory	12	35.3	27	15.3	0.50	4.074	<0.001
	Inflammatory	22	64.7	149	84.7			
Total protein	Below norms	2	6.1	22	12.6	−0.40	−2.162	0.031
	In norm	30	90.9	150	85.7			
	Above norms	1	3.0	3	1.7			

^1^ Classification based on Pourhassan et al. ^2^ Classification based on ESPEN. * Goodman Kruskal’s gamma correlation.
